# Supplementary material for: Taxonomic signatures of cause-specific mortality risk in human gut microbiome
Source: Nat Commun. 2021 May 11;12:2671. doi: 10.1038/s41467-021-22962-y (PMC8113604; doi:10.1038/s41467-021-22962-y)
Supplement: Supplementary file 1 — Supplementary Information [file 41467_2021_22962_MOESM1_ESM.docx]

**SUPPLEMENTARY INFORMATION**

**Taxonomic Signatures of Long-Term Mortality Risk in Human Gut Microbiome**

Aaro Salosensaari*^1,2,3^, Ville Laitinen*^2,3^, Aki S. Havulinna^4,5^, Guillaume Meric^,6,7^,

Susan Cheng^8,9^, Markus Perola^4^, Liisa Valsta^4^, Georg Alfthan^4^, Michael Inouye^6,7^,

Jeramie D. Watrous^10^, Tao Long^10^, Rodolfo A. Salido^11^, Karenina Sanders^11^, Caitriona Brennan^11^, Gregory C. Humphrey^11^, Jon G. Sanders^11^, Mohit Jain^10^, Pekka Jousilahti^4^,

Veikko Salomaa^4^, Rob Knight^11^, Leo Lahti*^2,3^, Teemu Niiranen*^1,4^

**SUPPLEMENTARY FIGURES**

####
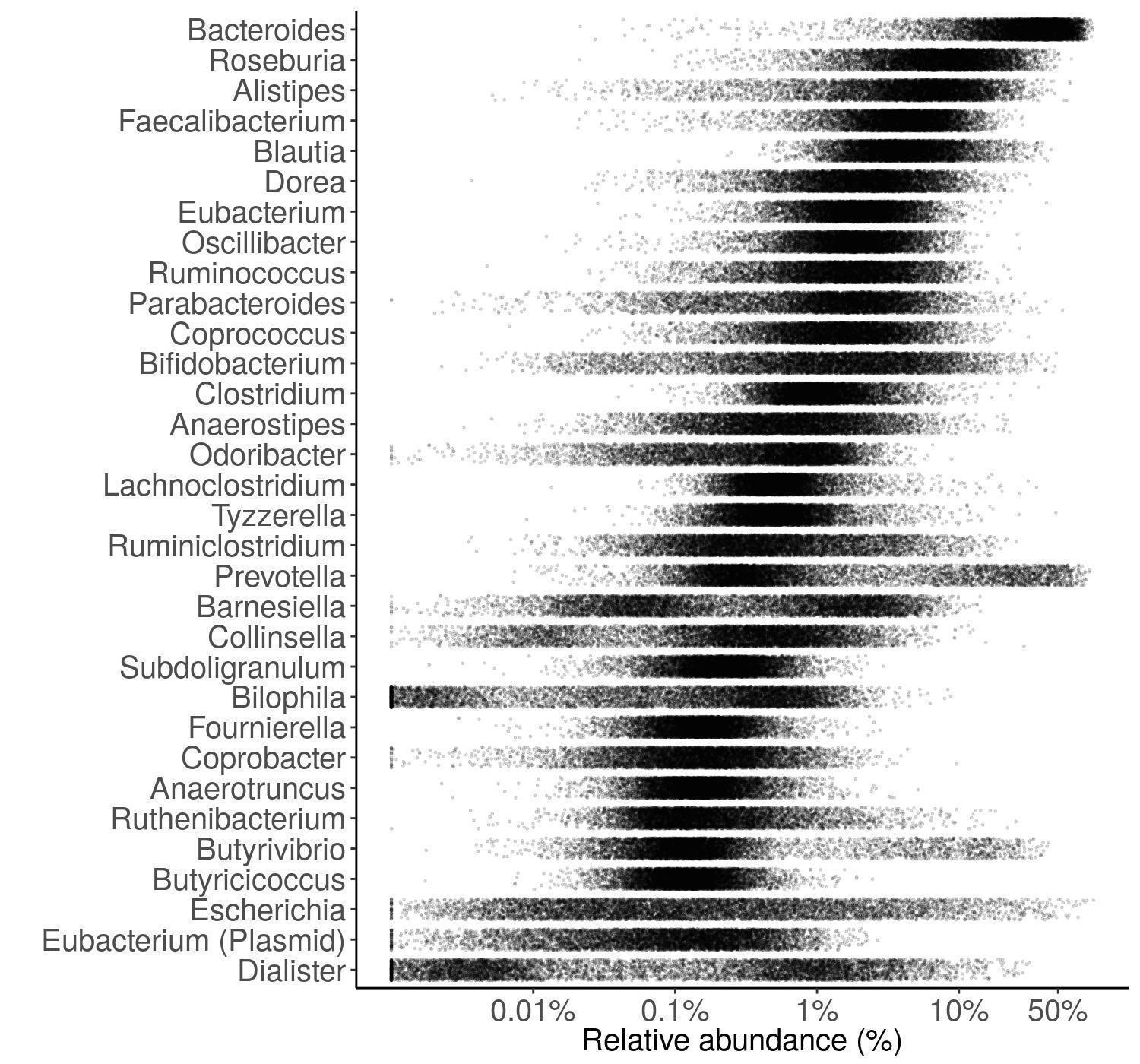


**Supplementary Fig. 1 Relative abundances for the 32 most abundant and prevalent genera that were detected at >0.1% relative abundance in the majority (>50%) of the study participants.** Each dot represents one individual, and the darker regions indicate more populated areas of the abundance landscape. On average, these genera cover 93.2% of the community based on their combined relative abundance.

####
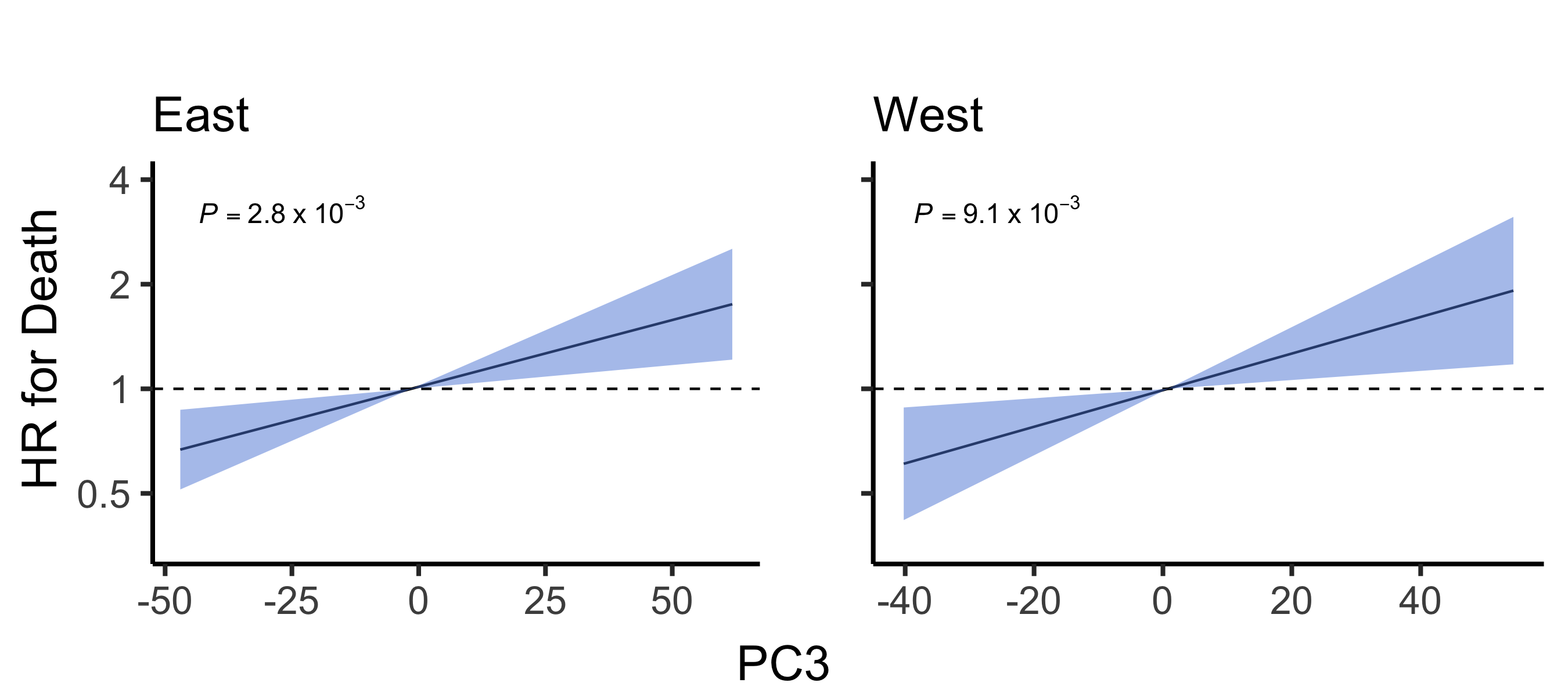


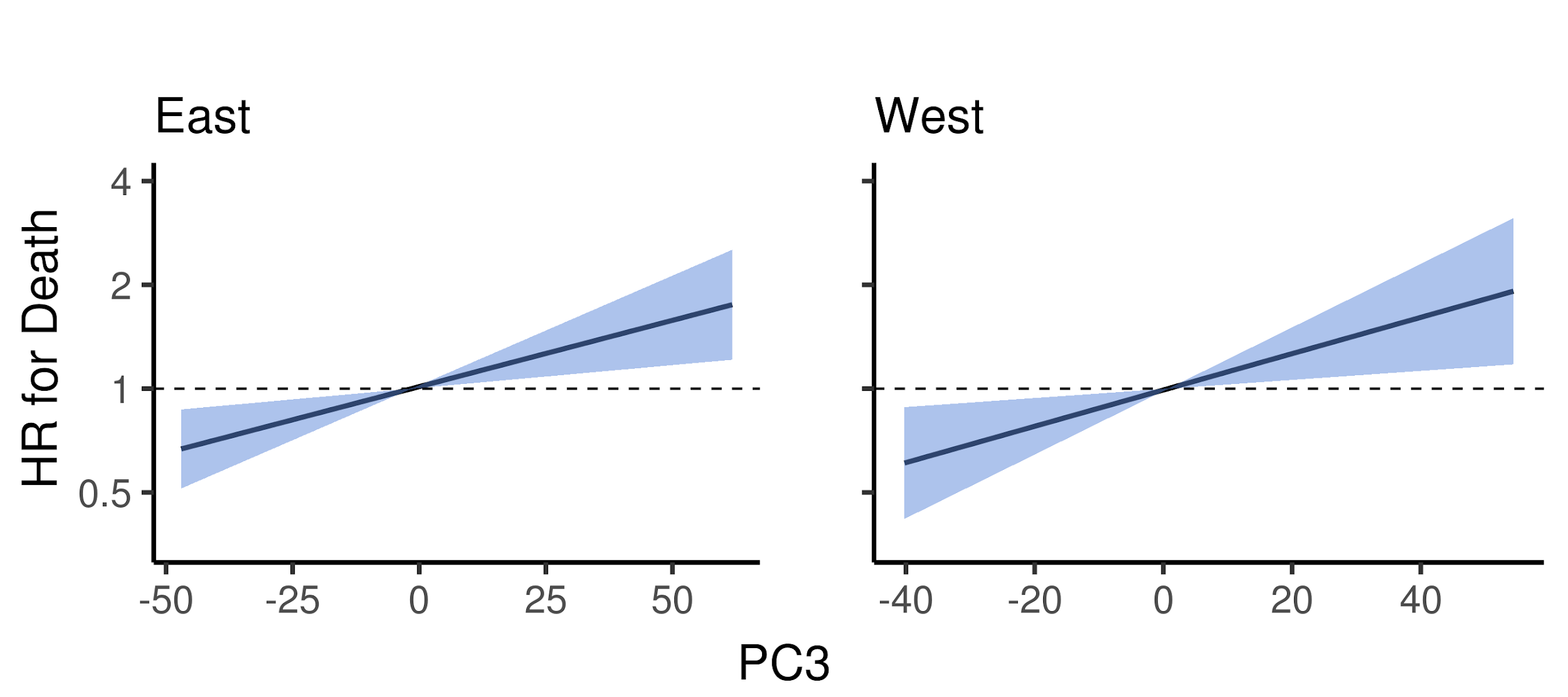


**Supplementary Fig. 2 Association between the third principal component (PC3) and mortality in the Eastern and Western Finnish populations.** Black line indicates the estimated hazard ratio compared to median PC3 values and blue area the 95% confidence interval (CI) of the hazard ratio. Unit variance increase in PC3 was related to hazard ratios of 1.14 (95% confidence interval [CI], 1.05–1.24; FDR-adjusted *P* = 2.8x10^-3^; two-tailed Wald test) and 1.190 (95% CI, 1.04–1.36; FDR-adjusted *P* = 9.1x10^-3^; two-tailed Wald test) in Eastern and Western Finnish populations, respectively. Analyses are adjusted for age, body mass index, sex, smoking, diabetes, use of antineoplastic and immunomodulating agents, systolic blood pressure and self-reported antihypertensive medication.

####
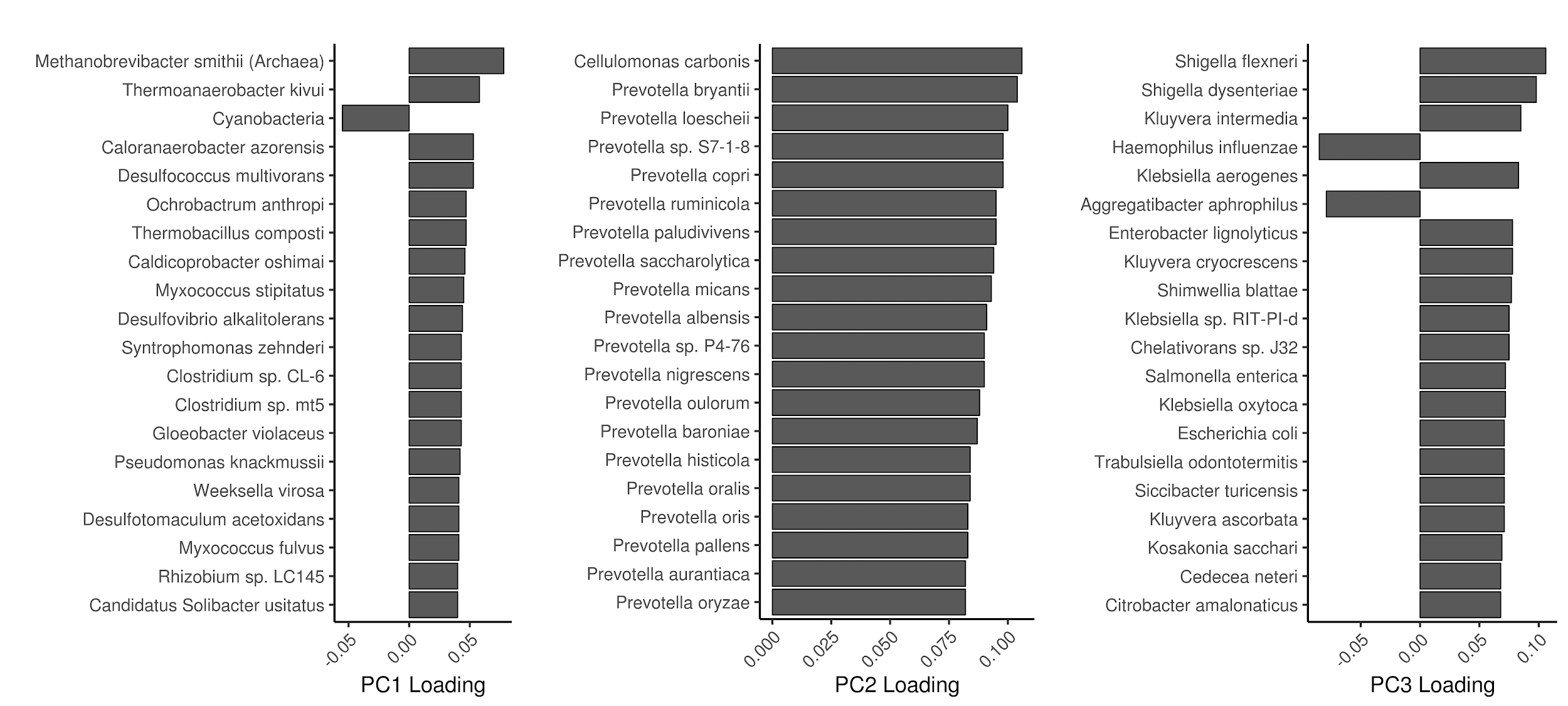


**Supplementary Fig. 3 Principal component driver species.** The 20 most important driver species of the first three principal components.

####
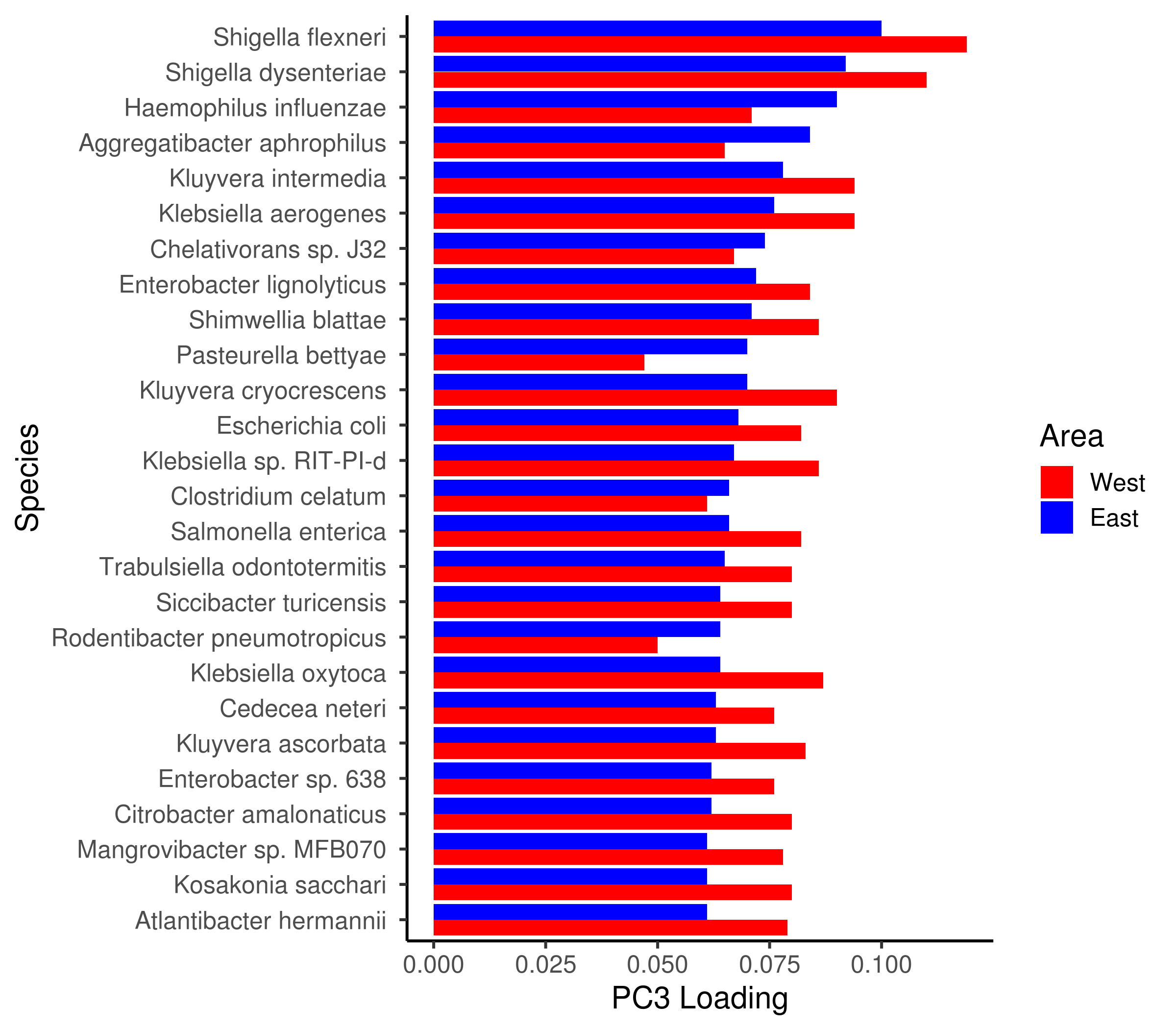


**Supplementary Fig. 4. Third principal component drivers in Eastern and Western Finnish populations.** Most important PC3 drivers in the Eastern and Western Finnish populations.


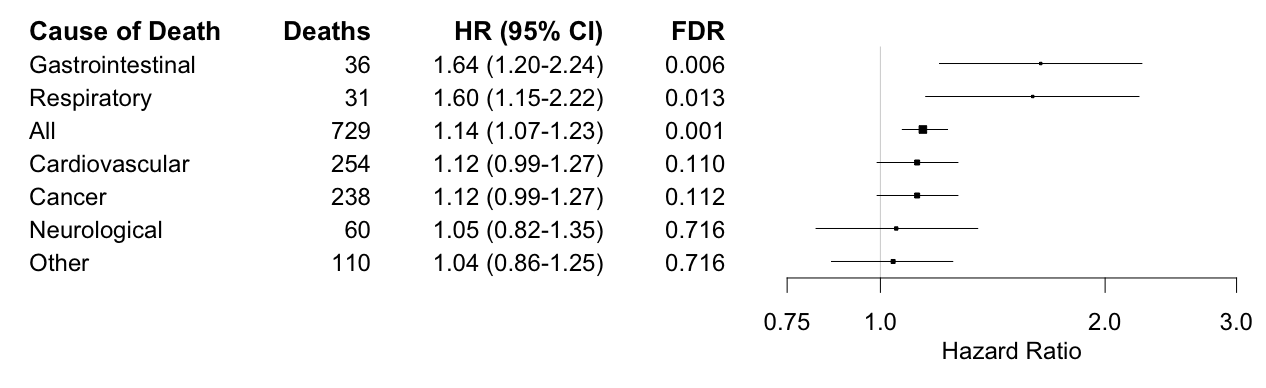


**Supplementary Fig. 5 The association between PC3 and cause-specific mortality.** Cox hazard ratios and 95% confidence intervals are reported per unit variance increase in the third principal component. Box sizes are inversely proportional to *P-*values. Entire study sample (*n* = 7211) was examined independently with each cause of death as end point. Analyses are adjusted for age, body mass index, sex, smoking, diabetes, use of antineoplastic and immunomodulating agents, systolic blood pressure and self-reported antihypertensive medication. HR, hazard ratio; FDR, false discovery rate.


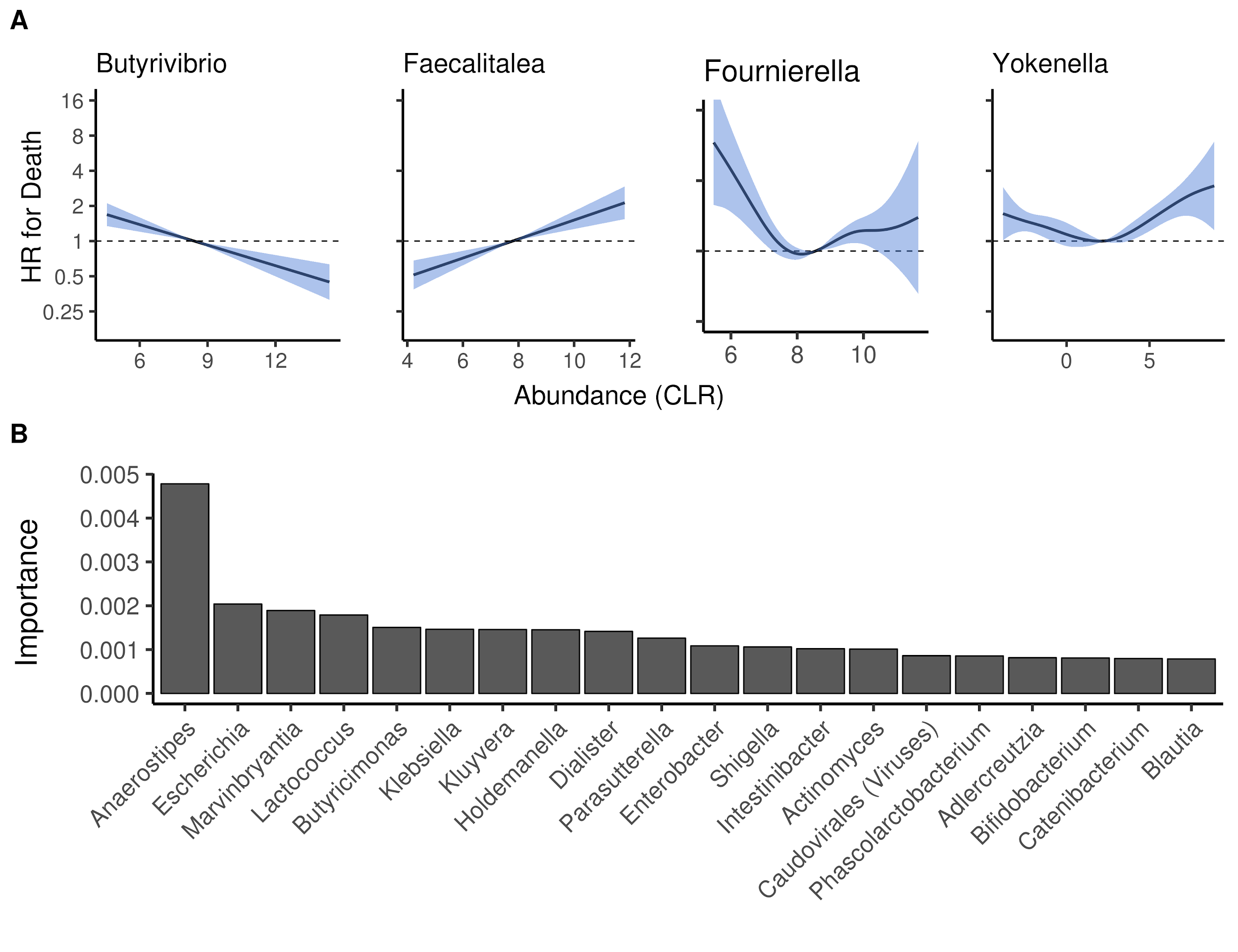


**Supplementary Fig. 6 Genus-level variation and mortality. A** Examples of observed linear and non-linear associations between bacterial abundances and mortality risk. Black line indicates the estimated hazard ratio compared to median abundance level and blue area indicates the 95% confidence interval of the hazard ratio (HR). **B** Importance scores for top 20 genera based on Random Survival Forest analysis. Analyses in panels A and B are adjusted for age, body mass index, sex, smoking, diabetes, use of antineoplastic and immunomodulating agents, systolic blood pressure and self-reported antihypertensive medication. Increased *Escherichia,* *Shigella* and *Kluyvera* were positively associated with mortality*, Parasutterella*  was negatively associated with mortality, while this relation was nonlinear for *Anaerostipes.*


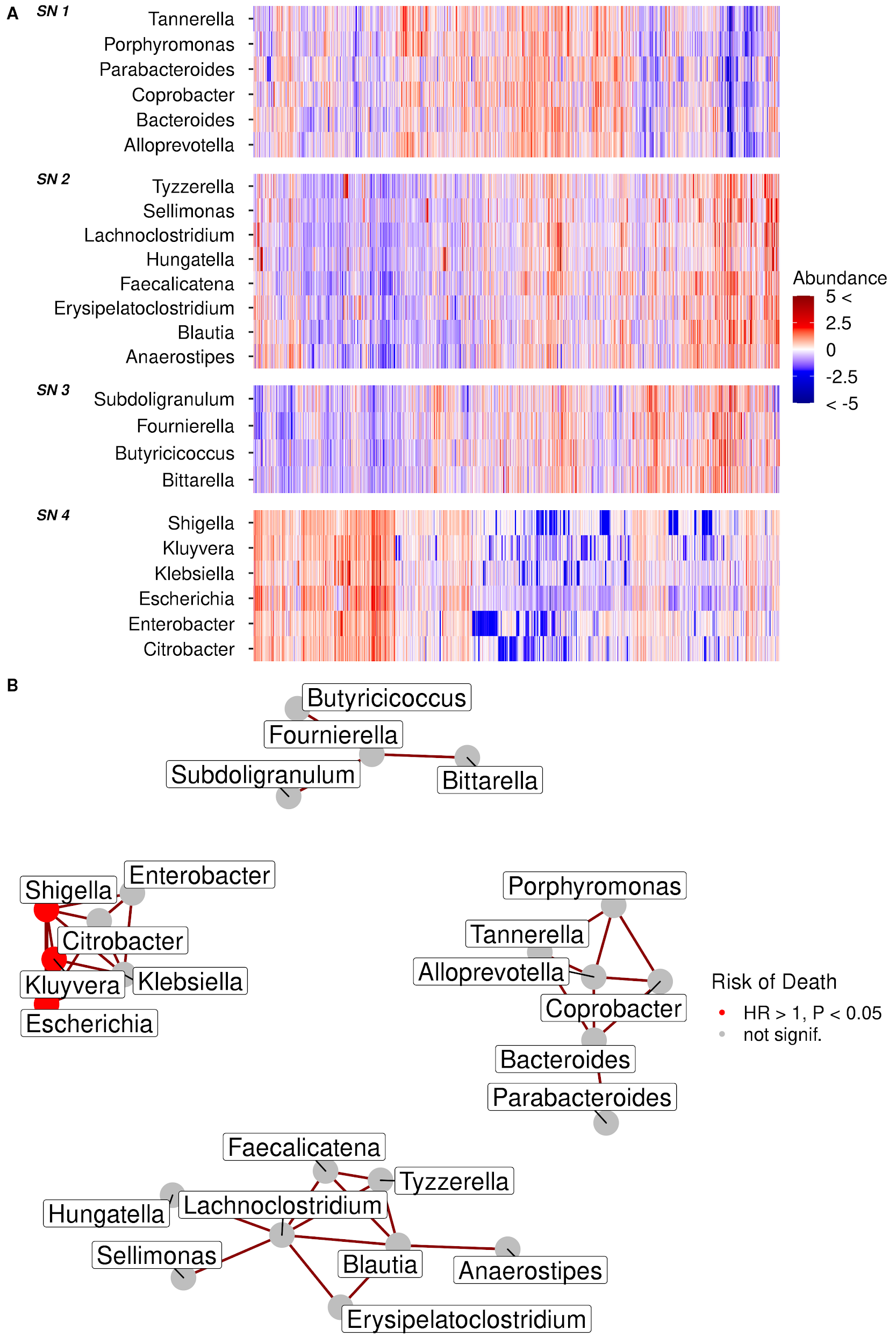


**Supplementary Fig. 7. The most robust taxonomic subnetworks and their association with mortality risk A**  Abundance variation across the study population in taxonomic subnetworks in panel B (CLR-transformed abundances centered at zero and scaled to unit variance). We ordered the samples with hierarchical clustering (Ward method) based on Spearman correlation of scaled and centered CLR-transformed abundances. **B** The observed network structure and the risk of death related to its components. HR, hazard ratio. Analyses are adjusted for age, body mass index, sex, smoking, diabetes, use of antineoplastic and immunomodulating agents, systolic blood pressure and self-reported antihypertensive medication; *P-*values are FDR-adjusted and based on the two-tailed Wald test. Subnetworks with only two or one genera have been excluded.

####
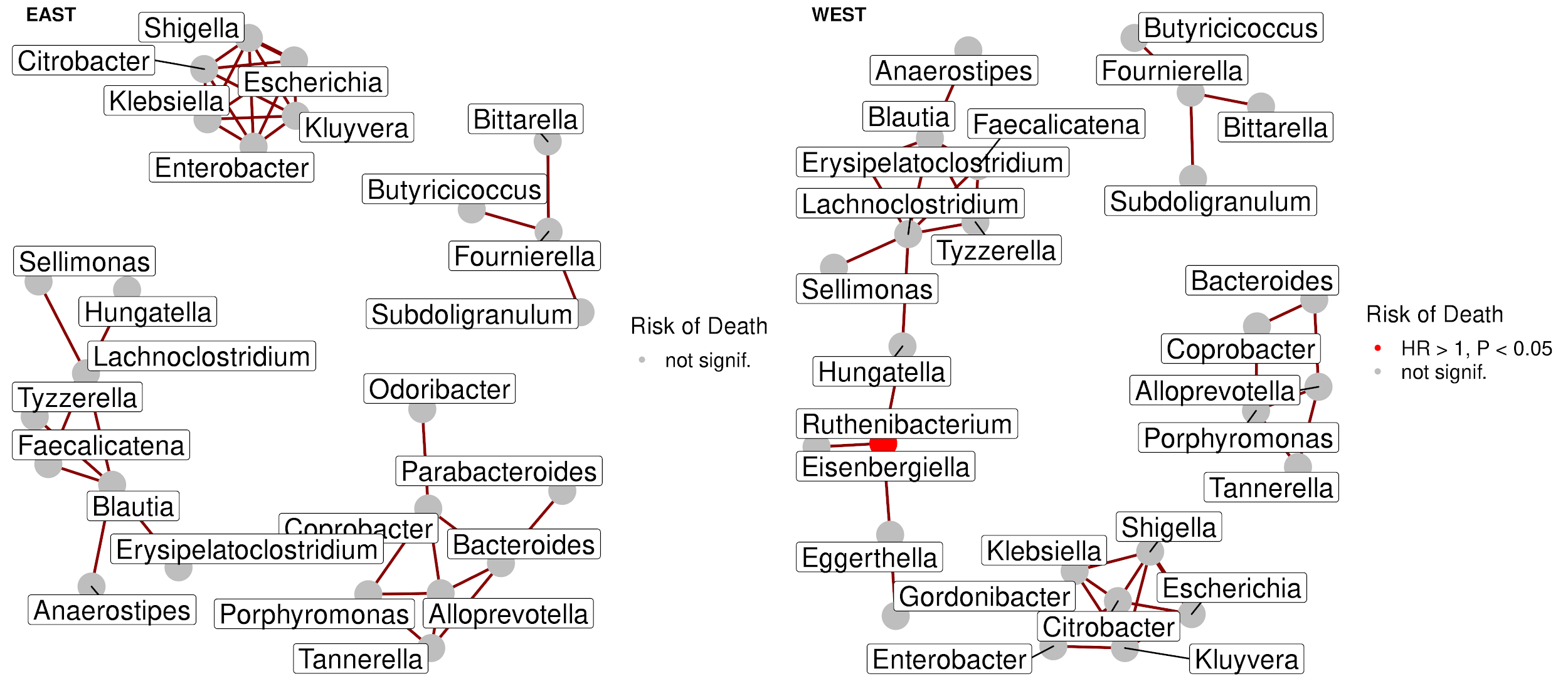


**Supplementary Fig. 8. Taxonomic subnetworks observed in the Eastern and Western Finnish populations** The mortality-associated subnetwork (*Escherichia, Shigella, Lambdavirus, Salmonella*, and others) can be observed both in the Eastern (*n*=4871; 519 deaths) and Western (*n* = 2184; 210 deaths) population with the same procedure as in the main analysis. Analyses are conducted separately for the Eastern and Western population and are adjusted for age, body mass index, sex, smoking, diabetes, use of antineoplastic and immunomodulating agents, systolic blood pressure and self-reported antihypertensive medication; *P-*values are FDR-adjusted and based on the two-tailed Wald test. Subnetworks with only two or one genera were excluded from further analysis.


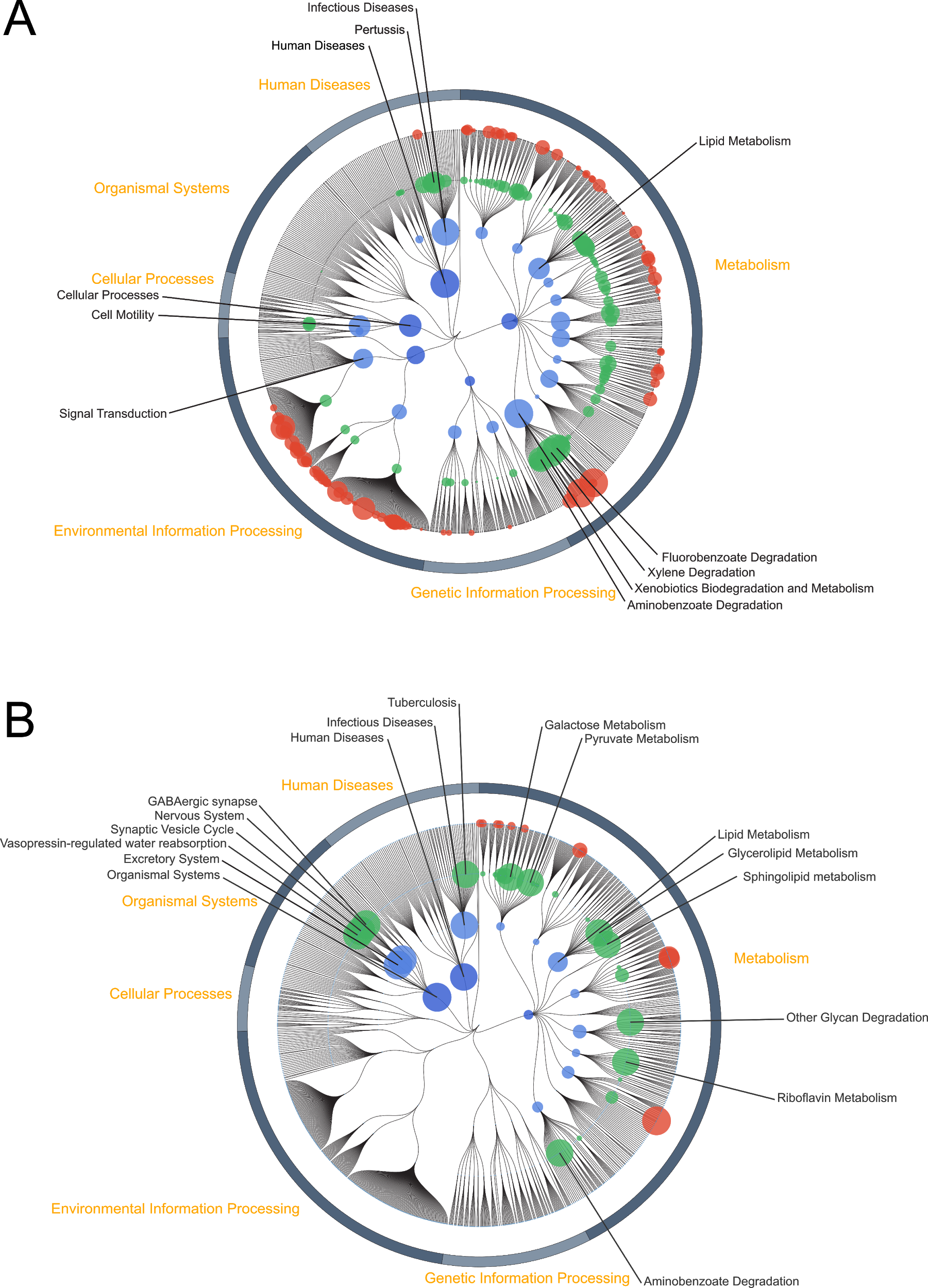


#### **Supplementary Fig. 9. Predicted functional pathways associated with (A) increased and (B) decreased mortality risk.** For the module (red), pathway (green), biological process (light blue), and biological category (dark blue) functional layers, node size corresponds to the average inverse *P-*value of the KEGG Orthology group assigned to that node. Only KO groups that were positively associated with mortality were included. Node titles are shown for nodes in the three highest layers with a size > 150.

#### **SUPPLEMENTARY TABLES**

**Supplementary Table 1.** Significant multivariable-adjusted relations of principal components 1–3 and alpha diversity with mortality (Cox proportional hazards based on two-tailed Wald test).

| **Predictor** | **Coefficient** | **SE** | **Hazard Ratio  (95% CI)** | **P (adjusted)** | **Test Statistics** | **Test** | **Association** |
| --- | --- | --- | --- | --- | --- | --- | --- |
| PC3 | 0.135 | 0.036 | 1.145 (1.066-1.229) | 0.00103 | 3.711 | Wald | linear |
| PC1 | -0.085 | 0.038 | 0.918 (0.852-0.99) | 0.06504 | -2.226 | Wald | linear |
| PC2 | -0.05 | 0.036 | 0.952 (0.886-1.022) | 0.17151 | -1.367 | Wald | linear |
| Shannon | -0.055 | 0.036 | 0.946 (0.881-1.016) | 0.17151 | -1.525 | Wald | linear |
| Observed | -0.053 | 0.038 | 0.948 (0.88-1.022) | 0.17151 | -1.398 | Wald | linear |

#### **Supplementary Table 2.** Associations of PC3 and covariates with mortality (Cox proportional hazards based on two-tailed Wald test).

| **Predictor** | **Coefficient** | **SE** | **Hazard Ratio  (95% CI)** | **P (adjusted)** | **Test Statistics** | **Test** | **Association** |
| --- | --- | --- | --- | --- | --- | --- | --- |
| Baseline Age | 1.34 | 0.06 | 3.816 (3.394-4.29) | <0.00001 | 22.393 | Wald | linear |
| Smoking | 0.99 | 0.09 | 2.682 (2.265-3.175) | <0.00001 | 11.451 | Wald | linear |
| Male sex | 0.67 | 0.08 | 1.954 (1.673-2.281) | <0.00001 | 8.473 | Wald | linear |
| Diabetes | 0.59 | 0.11 | 1.804 (1.451-2.243) | <0.00001 | 5.308 | Wald | linear |
| PC3 | 0.14 | 0.04 | 1.145 (1.066-1.229) | 0.00021 | 3.711 | Wald | linear |
| Systolic Blood Pressure | 0.13 | 0.04 | 1.138 (1.059-1.223) | 0.00045 | 3.508 | Wald | linear |
| Antineoplastic or immunomodulating agents | 0.75 | 0.26 | 2.109 (1.281-3.474) | 0.00336 | 2.933 | Wald | linear |
| BMI | 0.04 | 0.04 | 1.045 (0.964-1.134) | 0.28228 | 1.075 | Wald | linear |
| Antihypertensive Medication | 0.03 | 0.03 | 1.028 (0.966-1.093) | 0.38843 | 0.862 | Wald | linear |

#### **Supplementary Table 3.** Multivariable-adjusted linear (Cox proportional hazards based on Wald test) and non-linear (Chi-square test) associations between individual genera and mortality.

| **Predictor** | **Coefficient** | **SE** | **HR (95% CI)** | **P (adjusted)** | **Test Statistics** | **Test** | **Association** |
| --- | --- | --- | --- | --- | --- | --- | --- |
| *Butyrivibrio (Bacteria)* | −0.187 | 0.041 | 0.83 (0.765-0.899) | 0.003 | -4.524 | Wald | linear |
| *Ruthenibacterium (Bacteria)* | 0.166 | 0.037 | 1.181 (1.099-1.269) | 0.003 | 4.516 | Wald | linear |
| *Faecalitalea (Bacteria)* | 0.165 | 0.036 | 1.179 (1.099-1.264) | 0.003 | 4.598 | Wald | linear |
| *Cedecea (Bacteria)* | 0.168 | 0.035 | 1.183 (1.104-1.268) | 0.003 | 4.747 | Wald | linear |
| *Chromobacterium (Bacteria)* | −0.125 | 0.03 | 0.882 (0.831-0.937) | 0.011 | -4.116 | Wald | linear |
| *Candidatus Regiella (Bacteria)* | 0.138 | 0.034 | 1.148 (1.075-1.227) | 0.011 | 4.095 | Wald | linear |
| *Escherichia (Bacteria)* | 0.148 | 0.036 | 1.159 (1.08-1.244) | 0.011 | 4.103 | Wald | linear |
| *Planctopirus (Bacteria)* | −0.157 | 0.039 | 0.855 (0.792-0.922) | 0.012 | -4.028 | Wald | linear |
| *Scardovia (Bacteria)* | 0.139 | 0.036 | 1.149 (1.071-1.232) | 0.017 | 3.902 | Wald | linear |
| *Eggerthella (Bacteria)* | 0.148 | 0.038 | 1.16 (1.077-1.249) | 0.017 | 3.904 | Wald | linear |
| *Pluralibacter (Bacteria)* | 0.145 | 0.037 | 1.156 (1.074-1.244) | 0.017 | 3.87 | Wald | linear |
| *Succinimonas (Bacteria)* | −0.138 | 0.036 | 0.871 (0.811-0.935) | 0.019 | -3.802 | Wald | linear |
| *Atlantibacter (Bacteria)* | 0.136 | 0.036 | 1.146 (1.068-1.228) | 0.019 | 3.815 | Wald | linear |
| *Caldicoprobacter (Bacteria)* | −0.136 | 0.036 | 0.872 (0.812-0.937) | 0.021 | -3.746 | Wald | linear |
| *Syntrophomonas (Bacteria)* | −0.126 | 0.034 | 0.881 (0.824-0.942) | 0.021 | -3.712 | Wald | linear |
| *Parasutterella (Bacteria)* | −0.138 | 0.037 | 0.871 (0.81-0.937) | 0.021 | -3.726 | Wald | linear |
| *Arsenophonus (Bacteria)* | 0.126 | 0.034 | 1.134 (1.062-1.212) | 0.021 | 3.736 | Wald | linear |
| *Lachnospira (Bacteria)* | −0.129 | 0.035 | 0.879 (0.821-0.942) | 0.023 | -3.675 | Wald | linear |
| *Acetoanaerobium (Bacteria)* | −0.132 | 0.036 | 0.877 (0.816-0.942) | 0.024 | -3.617 | Wald | linear |
| *Sphingopyxis (Bacteria)* | −0.124 | 0.034 | 0.884 (0.827-0.944) | 0.024 | -3.646 | Wald | linear |
| *Leclercia (Bacteria)* | 0.129 | 0.036 | 1.138 (1.061-1.22) | 0.024 | 3.627 | Wald | linear |
| *Shimwellia (Bacteria)* | 0.13 | 0.036 | 1.138 (1.061-1.221) | 0.024 | 3.613 | Wald | linear |
| *Photorhabdus (Bacteria)* | 0.118 | 0.033 | 1.125 (1.055-1.2) | 0.027 | 3.571 | Wald | linear |
| *Shigella (Bacteria)* | 0.142 | 0.04 | 1.153 (1.066-1.247) | 0.028 | 3.553 | Wald | linear |
| *Faecalibacterium (Bacteria)* | −0.13 | 0.037 | 0.878 (0.816-0.944) | 0.033 | -3.498 | Wald | linear |
| *Tetrasphaera (Bacteria)* | 0.133 | 0.038 | 1.143 (1.06-1.232) | 0.035 | 3.463 | Wald | linear |
| *Pseudobutyrivibrio (Bacteria)* | −0.13 | 0.038 | 0.878 (0.815-0.946) | 0.035 | -3.428 | Wald | linear |
| *Erythrobacter (Bacteria)* | −0.122 | 0.036 | 0.885 (0.825-0.949) | 0.035 | -3.423 | Wald | linear |
| *Methylophilus (Bacteria)* | −0.133 | 0.039 | 0.876 (0.812-0.945) | 0.035 | -3.433 | Wald | linear |
| *Candidatus Ishikawaella (Bacteria)* | 0.108 | 0.032 | 1.114 (1.047-1.185) | 0.035 | 3.428 | Wald | linear |
| *Kluyvera (Bacteria)* | 0.126 | 0.037 | 1.135 (1.056-1.219) | 0.035 | 3.452 | Wald | linear |
| *Candidatus Koribacter (Bacteria)* | −0.123 | 0.036 | 0.884 (0.823-0.949) | 0.037 | -3.399 | Wald | linear |
| *Loktanella (Bacteria)* | −0.117 | 0.035 | 0.89 (0.831-0.953) | 0.046 | -3.33 | Wald | linear |
| *Thermaerobacter (Bacteria)* | −0.109 | 0.033 | 0.897 (0.841-0.957) | 0.048 | -3.31 | Wald | linear |
| *Yokenella (Bacteria)* |  |  |  | 0.001 | 33.176 | Chisq | non-linear |
| *Fournierella (Bacteria)* |  |  |  | 0.009 | 25.971 | Chisq | non-linear |
| *Arsenicicoccus (Bacteria)* |  |  |  | 0.027 | 22.963 | Chisq | non-linear |
| *Anaerostipes (Bacteria)* |  |  |  | 0.032 | 22.018 | Chisq | non-linear |
| *Trueperella (Bacteria)* |  |  |  | 0.038 | 20.706 | Chisq | non-linear |
| *Shuttleworthia (Bacteria)* |  |  |  | 0.038 | 20.887 | Chisq | non-linear |

**SUPPLEMENTARY METHODS**

**Statistical workflow for microbiome-based survival analysis**

We propose the following workflow to identify microbiome signatures with prospective time-to-event associations in population cohort studies. This can be used as the starting point to evaluate how strongly microbiome composition at a given time reflects later changes in health status over years or decades, and which microbiome features contribute most to such associations. The workflow covers standard aspects of taxonomic profiling studies, including alpha and beta diversity, differential abundance, co-occurrence networks, and functional data^1,2^ , including suggestions on specific methods that are widely adopted in contemporary microbiome research^1-3^ .

**1. Characterize the study cohort and the selected end point**

Total sample size; number and timing of events for the specified endpoint; relevant clinical and phenotypic metadata (**Fig. 1**).

**2. Unsupervised analysis of time-to-event associations**

Unsupervised analysis is used to quantify prospective associations of standard microbiome features that can be defined independently of the future end point.

Alpha diversity

Alpha diversity is a standard ecological index that quantifies the within-sample variation in relative species abundances.

- We recommend Shannon Index, estimated based on the full community profile at the finest taxonomic resolution (e.g. species level). This index is widely used in microbiome research, it gives weight also to less abundant community members, can be estimated without access to phylogenetic tree information, and is more robust to variations in sequencing read counts than other common measures^1^ . It is advisable to additionally verify that the main findings are robust to the choice of alpha diversity measure.

Beta diversity

Beta diversity is an ecological concept that quantifies the overall similarity between distinct communities, and it is regularly used to quantify variation in human microbiome composition between individuals at a given body site.

- Specific considerations: Following standard conventions in the field, we propose that beta diversity is estimated based on the full community profile at the finest taxonomic resolution (e.g. species level). We recommend the use of Bray-Curtis dissimilarity by default as it is a commonly used ecological beta diversity index, readily applicable to compositional data, and can be estimated without access to phylogenetic tree information. We recommend to additionally verify that the main findings are robust to the choice of dissimilarity measure.
- Visualization (**Fig. 1**): We recommend using Principal Coordinates Analysis (PCoA) to visualize beta diversity in order to facilitate comparability with other population studies where this method is a standard choice. As a non-linear technique it can capture a richer picture of the overall community variation than linear methods. Highlighting the most dominant community member in each individual can pinpoint specific community types or broad patterns of community variation.
- Association analysis (**Fig. 2**): whereas non-linear techniques are useful for intuitive visualization of similarities between high-dimensional microbial communities, we recommend the use of linear methods to quantify prospective associations between community variation and end points. Linear methods are potentially more robust to variations in the data and more straightforward to interpret. We recommend using Principal Component Analysis (PCA), which is one of the most commonly used linear ordination methods. It identifies interpretable patterns in microbial community composition that associate with the largest inter-individual differences. These broad population patterns can be linked to specific endpoints.

Abundance variation in specific community members

The analysis of the abundance of specific community members complements the analysis of overall community variation (alpha and beta diversity).

- We recommend the standard Cox proportional hazards model to quantify associations between the abundance of a given taxonomic group and time-to-event association with the given end point. The properties of this model are widely reported, and incorporation of covariate information is straightforward.
- Specific considerations: (i) Due to the compositional nature of microbiome data, we recommend applying the CLR transformation as a prior step before time-to-event analysis in order to remove compositionality bias^3^ . (ii) We propose that rare community members are excluded from the data before analysis as they may contain disproportionately high levels of taxonomic profiling noise^4^ . Hence, filtering the rare members can help to reduce the number of false positives in taxonomic profiling and considerably reduce multiple testing as most taxa are detected only at low abundance and prevalence. Whereas the specific thresholds may vary by study, we have excluded taxa that have <1% population prevalence at 0.1% abundance threshold. (iii) The time-to-event analysis can be performed at different taxonomic levels.

Abundance variation in taxonomic co-occurrence networks

Analysis of individual community members can be complemented by investigating specific sub-communities. In unsupervised analysis, we start by identifying robust sets of frequently co-occurring community members, and then quantifying their prospective associations with the specific end points.

- We recommend using SPIEC-EASI to reconstruct taxonomic co-occurrence networks. This method is widely adopted in microbiome research , it is robust to compositionality effects^3^ , scalable to large-scale population studies, and returns a sparse network, thus enhancing interpretability and robustness. A specific threshold can be chosen to recover the most robust subnetworks.
- Cox proportional hazards model can be used to quantify prospective association in subnetwork abundance (total combined abundance of the subnetwork members) and the given end point. The results obtained with subnetworks can be readily compared to those obtained with individual community members (see above).

Functional data

Functional data is often available as predictions based on the taxonomic composition of the community from standard microbiome bioinformatics pipelines but the recommendations apply also to measured functions, e.g. metabolomic measurements.

- We recommend the use of Cox proportional hazards model also for quantifying univariate associations between the abundance or activity of specific community functions and the given end points. The results can be directly compared with the taxonomic groups and subnetworks (see above).
- Specific considerations: the functional data can be statistically transformed before analysis in order to reduce skewness and obtain more normally distributed data.

**3. Supervised analysis**

Supervised analysis is used to specifically identify the microbiome signatures that are most strongly associated with the selected end points. This complements the analysis of standard community features that can be defined independently of the time-to-event association.

- We recommend using Random Survival Forests^5^ (RSF) for robust feature selection in supervised time-to-event analysis. This allows one to identify the set of community members that collectively exhibits the strongest association with the given end point. The non-parametric RSF has certain practical advantages: it is less sensitive to the distributional properties and outliers and less prone to overfitting than the classical model-based approaches^6^ ). Random Forest-based feature selection has been also recently recommended more generally among best practices in microbiome profiling studies^1^ .

**4. Sensitivity analysis and replication**

The sensitivity of the observed associations can be estimated based on a combination of standard techniques, and further validated in external data sets.

- All analyses should be controlled for the known relevant covariates, such as sex, age, body-mass index, diet and medications
- The sensitivity of the results can be further analyzed by investigating the reproducibility of the results in sample subsets that represent for instance geographically distinct populations or excluding individuals with specific medications (e.g. antibiotics).
- Internal cross-validation can be used to assess generalizability to new data; ideally, the cross-validation can consider subsets that have distinct populations for instance based on different geographical or cultural groupings.
- It is recommended to test the main findings in an external replication cohort, when available.

**Healthy Food Choices Score**

Habitual diet was assessed using a food propensity questionnaire (FPQ) which contained 42 food items and had choices ranging from 1 – 6 for consumption frequency (see: item 101 in https://thl.fi/documents/189940/4850942/FINRISKI2002questionnaireENGLISH.pdf/dc27a910-80aa-4bfa-bf08-a59051caadd9). Answers were converted to times-per-month values in the following way: An answer 1 (“Less than once a month”) was converted to 0.5 times per month, 2 (“Once or twice a month”) to 1.5 times per month, 3 (“Once a Week”) to 4.3 times per month, 4 (“Couple of times a week”) to 8.6 times per month, 5 (“Almost every day”) to 21.5 times per month, and finally 6 (“Once a day or more often”) to 30, 45 or 60 times per month. Food items that are rarely eaten more than once a day such as pizzas, hamburgers, ice cream etc. were given the value of 30 times per month. Food items that are often eaten multiple times a day such as fresh vegetables, fruit, cheeses, breads, etc. were given a value of 60 times per month. Food items that fall in between these two groups were given 45 points.

A Healthy Food Choices (HFC) score was formed by choosing and combining FPQ-responses to food items that are recommended in the Nordic Nutrition Recommendations dietary guidelines to be part of a healthy diet^7^ . The HFC score and our use of the term ‘healthy’ encompasses some but not all characteristics of these guidelines. Food items chosen to be components of the score were fiber-rich breads, vegetables (incl. beans and lentils), fruits, berries, fresh berry and fruit juices, fish, poultry, low-fat cheeses, salad dressings and oils, and nuts and seeds. The HFC score does not take into consideration salt intake, usage of red and processed meat or liquid dairy products. As such, the HFC score simulates a diet rich in plants, fiber and unsaturated fatty acids.

The HFC score was calculated by summing the transformed monthly consumption scores for all chosen components. Additionally, a summary variable for total fiber was created, which combined scores of fiber-rich breads, vegetables, fruits, berries, and fresh fruit and berry juices into one. A summary of the HFC score’s structure and a listing of each components’ respective constituting food items are displayed below in the table along with their possible score ranges.

**Summary of the HFC score’s components, their food items and score ranges.**

| Components | Constituting food items | Score range^1^ |
| --- | --- | --- |
| Breads | Rye- and crisp bread^2^ | 1 – 120 |
|  | Graham- and multi-grain bread^2^ |  |
| Vegetables | Fresh vegetables and root vegetables^2^ | 1.5 – 150 |
|  | Cooked vegetables, beans and legumes^3^ |  |
|  | Vegetable dishes^3^ |  |
| Fruits | Fruits^2^ | 0.5 – 60 |
| Berries | Fresh and frozen berries^3^ | 0.5 – 45 |
| Juices | Fruit and berry juices^3^ | 0.5 – 45 |
| Fish | Fish, fish products and fish dishes^3^ | 0.5 – 45 |
| Poultry | Poultry, poultry products and poultry dishes^3^ | 0.5 – 45 |
| Low-fat cheeses | Low-fat cheeses^2^ | 0.5 – 60 |
| Dressings and oils | Salad dressings and oils^3^ | 0.5 – 45 |
| Nuts and seeds | Nuts^3^ | 1 – 90 |
|  | Seeds^3^ |  |

^1^Each component’s possible range displayed in times-per-month values. Individual food items’ score range for those items marked with^2^ is 0.5 – 60 and for those marked with^3^ it is 0.5 – 45 points.

**References**

1. Knight, R., et al*.* Best practices for analysing microbiomes. *Nat Rev. Microbiol.* **16,** 410-422 (2018).

2. Callahan, B. J., Sankaran K., Fukuyama J. A., McMurdie, P. J. & Holmes, S. P.*.* Bioconductor Workflow for Microbiome Data Analysis: from raw reads to community analyses. *F1000Research* **5,** 1492 (2016).

3. Gloor, G. B., Macklaim, J. M., Pawlowsky-Glahn, V., & Egozcue, J. J. Microbiome Datasets Are Compositional: And This Is Not Optional. *Front. Microbiol.* ***8,*** 2224 (2017).

4. Ye, S. H., Siddle, K. J., Park, D. J., Sabeti, P. C. Benchmarking Metagenomics Tools for Taxonomic Classification. *Cell* **178,** 779-794 (2019).

5. Ishwaran, H., Kogalur, U. B., Blackstone, E. H. & Lauer, M. S. Random survival forests. *Ann. Appl. Stat.* **2** (**3*),*** 841–860 (2008).

6. Dietrich S., et al*.* Random Survival Forest in practice: a method for modelling complex metabolomics data in time to event analysis. *Int, J, of Epidemiol.* **45,**1406-1420 (2016).

7. Nordic Council of Ministers, Nordic Nutrition Recommendations 2012 - Integrating nutrition and physical activity, 5th ed., Nordisk Ministerråd (2014)
