# Supplementary material for: Taxonomic signatures of cause-specific mortality risk in human gut microbiome
Source: Nat Commun. 2021 May 11;12:2671. doi: 10.1038/s41467-021-22962-y (PMC8113604; doi:10.1038/s41467-021-22962-y)
Supplement: Supplementary file 3 — Description of Additional Supplementary Files [file 41467_2021_22962_MOESM3_ESM.docx]

**Description of Additional Supplementary Files**

**File Name**: Supplementary Data 1
**Description:** Distribution of phylum relative abundance and prevalence in FINRISK 2002.

**File Name:** Supplementary Data 2
**Description:** Distribution of genus relative abundance and prevalence in FINRISK 2002.

**File Name**: Supplementary Data 3
**Description:** The top 20 drivers of the principal components 1– 3.

**File Name**: Supplementary Data 4
**Description:** Prevalence of the VFDB virulence genes among the FINRISK samples.

**File Name**: Supplementary Data 5
**Description:** Importance scores for genus abundances and covariates in Random Survival Forest.

**File Name:** Supplementary Data 6
**Description:** Taxonomic co-occurrence network estimated with SPIEC-EASI.

**File Name:** Supplementary Data 7
**Description:** Multivariable-adjusted associations between KEGG Orthology groups and mortality (Cox proportional hazards based on two-tailed Wald test).
