# Supplementary material for: Taxonomic signatures of cause-specific mortality risk in human gut microbiome
Source: Nat Commun. 2021 May 11;12:2671. doi: 10.1038/s41467-021-22962-y (PMC8113604; doi:10.1038/s41467-021-22962-y)
Supplement: Supplementary file 11 — Reporting Summary [file 41467_2021_22962_MOESM11_ESM.pdf]

nature research

Corresponding author(s): Teemu Niiranen (teemu.niiranen@utu.fi)
Leo Lahti (leo.lahti@utu.fi)

Last updated by author(s): Apr 29, 2020

Reporting Summary

Nature Research wishes to improve the reproducibility of the work that we publish. This form provides structure for consistency and transparency in reporting. For further information on Nature Research policies, see Authors & Referees and the Editorial Policy Checklist .

Statistics

For all statistical analyses, confirm that the following items are present in the figure legend, table legend, main text, or Methods section.

n/a	Confirmed	
☐	☒	The exact sample size (*n*) for each experimental group/condition, given as a discrete number and unit of measurement
☐	☒	A statement on whether measurements were taken from distinct samples or whether the same sample was measured repeatedly
☐	☒	The statistical test(s) used AND whether they are one- or two-sided<br>*Only common tests should be described solely by name; describe more complex techniques in the Methods section.*
☐	☒	A description of all covariates tested
☐	☒	A description of any assumptions or corrections, such as tests of normality and adjustment for multiple comparisons
☐	☒	A full description of the statistical parameters including central tendency (e.g. means) or other basic estimates (e.g. regression coefficient) AND variation (e.g. standard deviation) or associated estimates of uncertainty (e.g. confidence intervals)
☐	☒	For null hypothesis testing, the test statistic (e.g. *F*, *t*, *r*) with confidence intervals, effect sizes, degrees of freedom and *P* value noted<br>*Give P values as exact values whenever suitable.*
☒	☐	For Bayesian analysis, information on the choice of priors and Markov chain Monte Carlo settings
☒	☐	For hierarchical and complex designs, identification of the appropriate level for tests and full reporting of outcomes
☒	☐	Estimates of effect sizes (e.g. Cohen's *d*, Pearson's *r*), indicating how they were calculated

Our web collection on statistics for biologists contains articles on many of the points above.

Software and code

Policy information about availability of computer code

Data collection	*Provide a description of all commercial, open source and custom code used to collect the data in this study, specifying the version used OR state that no software was used.*
Data analysis	Published software applications, libraries and code used: Atropos (for sequence trimming), Bowtie2 on GRCh38 (for host read removal), Shogun v1.0.5 with NCBI RefSeq v82 (for taxonomic assignment and functional profiling), Anvi'o v5.5 and samtools v1.9 (for virulence gene analysis), FuncTree (for functional analysis and visualization). Custom R code (for taxonomic and mortality analysis) which utilizes libraries phyloseq, SpiecEasi, survival, randomForestSRC is shared as a supplemental file.

For manuscripts utilizing custom algorithms or software that are central to the research but not yet described in published literature, software must be made available to editors/reviewers. We strongly encourage code deposition in a community repository (e.g. GitHub). See the Nature Research guidelines for submitting code & software for further information.

Data

Policy information about availability of data

All manuscripts must include a data availability statement. This statement should provide the following information, where applicable:

- Accession codes, unique identifiers, or web links for publicly available datasets
- A list of figures that have associated raw data
- A description of any restrictions on data availability

The data used in this study are available from the THL Biobank upon submission of a research plan and signing a data transfer agreement (https://thl.fi/en/web/thl-biobank/for-researchers/application-process). The data are not openly available as they contain sensitive information from healthcare registers.

Field-specific reporting

Please select the one below that is the best fit for your research. If you are not sure, read the appropriate sections before making your selection.

- [x] Life sciences
- [ ] Behavioural & social sciences
- [ ] Ecological, evolutionary & environmental sciences

For a reference copy of the document with all sections, see nature.com/documents/nr-reporting-summary-flat.pdf

Life sciences study design

All studies must disclose on these points even when the disclosure is negative.

Sample size	No sample size calculations were performed, as the primary objective of the study was assessment of cardiovascular health in the Finnish population. The sample size was mainly determined by the availability of resources.
Data exclusions	Samples from 20 subjects were excluded because of total sample readcount was low (< 50 000), yielding n=7211 available for unsupervised analysis. For survival analysis of mortality, further 156 were excluded, as only 7055 participants had the full covariate information available (BMI, systolic blood pressure, smoking, antihypertensive medication use, diabetes status, use of antineoplastic or immunomodulating agents).
Replication	After contacting several groups with large-scale microbiome data, we were unable to find cohorts with similar prospective outcome data as in our study. For validation, we split the cohort into two subsamples according to geographic regions (Eastern Finns, n=4979 vs Western Finns, n=2184) with differing genetic backgrounds, lifestyles, and mortality rates. The then assessed the association between our main exposure (PC3) and mortality in both subsamples. As an additional analysis, we identified mortality-associated microbiome features in the Eastern population based on the Random Survival Forest model, and then tested their performance in the Western population.
Randomization	Participants were not randomly allocated to experimental groups. The study was an observational study based on a stratified random sample of the Finnish population aged 25-74 years from a several geographical areas. The dates and causes of deaths (the outcome under study, n=729) were obtained from the National Causes-of-Death register. The relevant covariates were adjusted for in our statistical analyses using data collected by a nurse during the baseline examination (BMI, systolic blood pressure), participants' self-reported answers to questionnaire (smoking, antihypertensive medication use) and national registers when such information was available (diabates from the Hospital Discharge Register and the Drug Reimbursement Register; use of antineoplastic or immunomodulating agents from the Drug Purchase Register).
Blinding	Issue of blinding was not relevant, as study had no expirimental group allocation.

Reporting for specific materials, systems and methods

We require information from authors about some types of materials, experimental systems and methods used in many studies. Here, indicate whether each material, system or method listed is relevant to your study. If you are not sure if a list item applies to your research, read the appropriate section before selecting a response.

Materials & experimental systems

n/a	Involved in the study	
[x]	[ ]	Antibodies
[x]	[ ]	Eukaryotic cell lines
[x]	[ ]	Palaeontology
[x]	[ ]	Animals and other organisms
[ ]	[x]	Human research participants
[x]	[ ]	Clinical data

Methods

n/a	Involved in the study	
[x]	[ ]	ChIP-seq
[x]	[ ]	Flow cytometry
[x]	[ ]	MRI-based neuroimaging

Human research participants

Policy information about studies involving human research participants

Population characteristics	The random stratified survey sample (stratified by sex, region and 10-year age group, based on information from the National Population Information System that includes information on all individuals living in Finland.) was drawn from original population sample of 13500 from several geographical areas in Finland. The overall participation rate was 65.5% (n=8798), and 7211 provided stool samples that were sequenced and used in analyses. 7055 participants had a full covariate information for mortality analysis, with mean age of 49 years, 55% being women and mean BMI of 27. In addition to age, BMI and systolic BP, other covariates considered were smoking (n=1648), diabetes (n=401), antihypertensive medication (n=1096), and use of antineoplastic or immunomodulating agents (n=62).
Recruitment	Participants from the original sample (as determined by stratified sampling, see above) were invited to participate in the study with a letter in mail. Although the overall participation rate was high, the sample may be subject to selection bias.
Ethics oversight	The study was approved by the Coordinating Ethical Committee of the Helsinki and Uusimaa Hospital District (Ref. 558/E3/2001)

Note that full information on the approval of the study protocol must also be provided in the manuscript.
